# Supplementary material for: The Natural History of CNGB1-Related Retinopathy: A Longitudinal Phenotypic Analysis
Source: Int J Mol Sci. 2022 Jun 17;23(12):6785. doi: 10.3390/ijms23126785 (PMC9245601; doi:10.3390/ijms23126785)
Supplement: Supplementary file 1 [file ijms-23-06785-s001.zip › ijms-1758271 Supplementary figure.pdf]

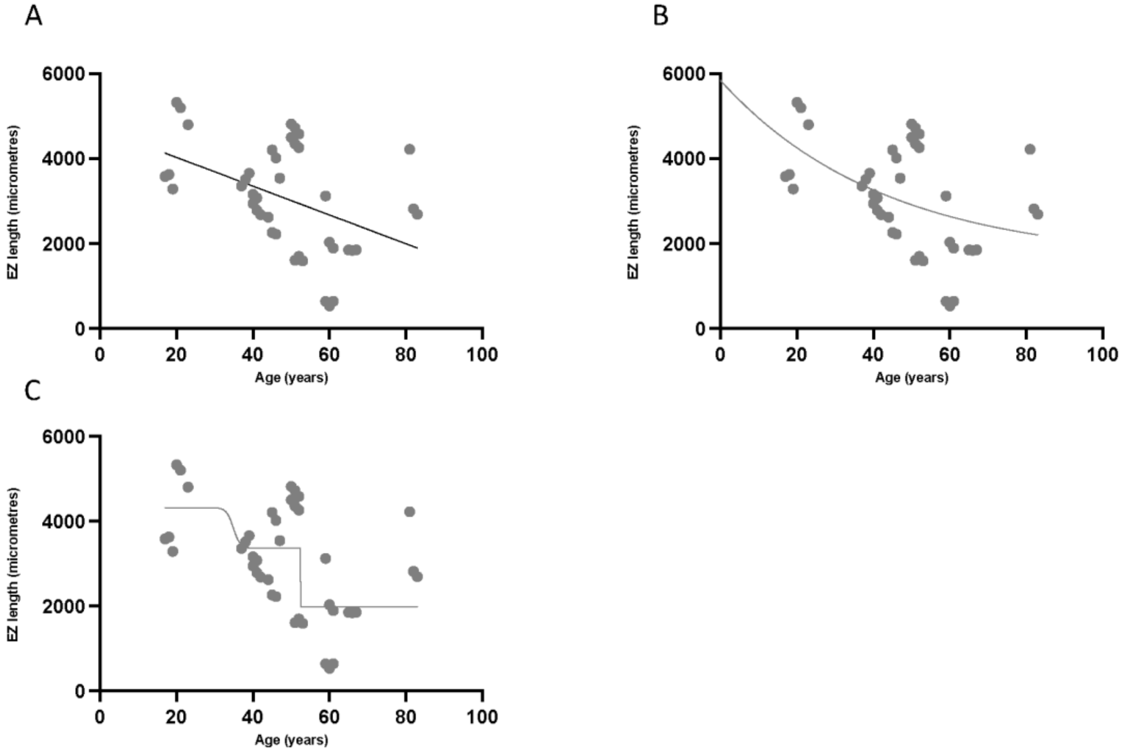

Figure S1. Ellipsoid zone length analysis with linear (A), exponential (B) and logarithmic (C) trends fitted. The relationship between ellipsoid zone length and age was best represented with a linear or exponential trend.
